# Mixology of MA_1–*x*_EA_*x*_PbI_3_ Hybrid Perovskites: Phase Transitions, Cation Dynamics, and Photoluminescence

**DOI:** 10.1021/acs.chemmater.2c02807

**Published:** 2022-11-02

**Authors:** Mantas Šimėnas, Sergejus Balčiu̅nas, Anna Ga̧gor, Agnieszka Pienia̧żek, Kasper Tolborg, Martynas Kinka, Vytautas Klimavicius, Šaru̅nas Svirskas, Vidmantas Kalendra, Maciej Ptak, Daria Szewczyk, Artur P. Herman, Robert Kudrawiec, Adam Sieradzki, Robertas Grigalaitis, Aron Walsh, Mirosław Ma̧czka, Ju̅ras Banys

**Affiliations:** † Faculty of Physics, Vilnius University, Sauletekio 3, LT-10257Vilnius, Lithuania; ‡ Institute of Low Temperature and Structure Research, Polish Academy of Sciences, Okólna 2, 50-422, PL-50-422Wroclaw, Poland; § Department of Semiconductor Materials Engineering, Wroclaw University of Science and Technology, Wybrzeze Wyspianskiego 27, PL-50-370Wroclaw, Poland; ∥ Thomas Young Centre and Department of Materials, Imperial College London, SW7 2AZLondon, U.K.; ⊥ Institute of Chemical Physics, Vilnius University, Sauletekio 3, LT-10257Vilnius, Lithuania; # Department of Experimental Physics, Wroclaw University of Science and Technology, Wybrzeze Wyspianskiego 27, PL-50-370Wroclaw, Poland

## Abstract

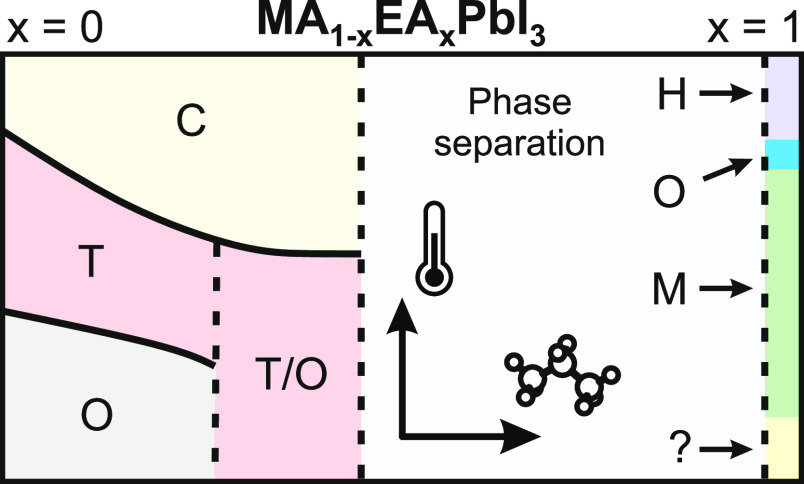

Mixing molecular cations in hybrid lead halide perovskites is a highly effective approach to enhance the stability and performance of optoelectronic devices based on these compounds. In this work, we prepare and study novel mixed 3D methylammonium (MA)–ethylammonium (EA) MA_1–*x*_EA_*x*_PbI_3_ (*x* < 0.4) hybrid perovskites. We use a suite of different techniques to determine the structural phase diagram, cation dynamics, and photoluminescence properties of these compounds. Upon introduction of EA, we observe a gradual lowering of the phase-transition temperatures, indicating stabilization of the cubic phase. For mixing levels higher than 30%, we obtain a complete suppression of the low-temperature phase transition and formation of a new tetragonal phase with a different symmetry. We use broad-band dielectric spectroscopy to study the dielectric response of the mixed compounds in an extensive frequency range, which allows us to distinguish and characterize three distinct dipolar relaxation processes related to the molecular cation dynamics. We observe that mixing increases the rotation barrier of the MA cations and tunes the dielectric permittivity values. For the highest mixing levels, we observe the signatures of the dipolar glass phase formation. Our findings are supported by density functional theory calculations. Our photoluminescence measurements reveal a small change of the band gap upon mixing, indicating the suitability of these compounds for optoelectronic applications.

## Introduction

Hybrid methylammonium (MA, CH_3_NH_3_^+^) lead halide perovskites MAPbX_3_ (X = I, Br, and Cl) are extensively investigated as efficient and solution-processable photovoltaic materials.^1,2^ The power conversion efficiency of solar cells based on these compounds experienced an extraordinary boost in the last decade and currently exceeds 25%.^3−8^ Several key physical properties such as the large absorption coefficient,^9^ optimal band gap,^10^ long carrier diffusion length,^11,12^ low exciton binding energy,^13^ and defect tolerance^14^ are responsible for the high performance of these materials. Some of these properties and thus the device operation can be significantly affected by the dynamics of molecular cations and structural phase transitions occurring in these compounds.^10,15−20^

The best-performing photovoltaic devices based on hybrid perovskites are obtained using compositions with mixed A-site cations.^21,22^ The most popular alternatives to MA are formamidinium (FA, HC(NH_2_)_2_^+^)^7,8^ and Cs^+^,^23^ although others such as ethylammoniumm (EA, CH_3_CH_2_NH_3_^+^),^24−29^ dimethylammonium (DMA, (CH_3_)_2_NH_2_^+^),^30−32^ methylhydrazinium (MHy, CH_3_NH_2_NH_2_^+^),^33,34^ and Rb^+^^35^ are also becoming of great interest. In addition to the improved performance, cation mixing significantly increases the stability of lead halide perovskites.^22^ For example, mixing prevents a spontaneous conversion of the desirable photoactive black phase of FAPbI_3_ and CsPbI_3_ to the photoinactive yellow phase.^36−38^

Despite many studies concentrating on the photovoltaic performance of mixed lead halide perovskites, mixing effects on the structural phases, phase transitions, and cation dynamics are significantly less studied and understood. Previously, we investigated these phenomena in MA_1–*x*_DMA_*x*_PbBr_3_^32^ and MA_1–*x*_FA_*x*_PbBr_3_^39^ compounds, revealing stabilization of the desirable cubic phase by suppression of the structural phase transitions. In addition, strong signatures of the electric dipole glass phase resulting from the frustrated MA cations were observed in MA_1–*x*_DMA_*x*_PbBr_3_,^32^ while this effect was weaker in MA_1–*x*_FA_*x*_PbBr_3_ mixed systems.^39^ Indications of the dipolar glass phase were also previously observed in MA_1–*x*_Cs_*x*_PbBr_3_^40^ and MA_1–*x*_FA_*x*_PbI_3_^41^ mixtures.

In this work, we use a suite of experimental and theoretical techniques to study a promising novel mixed-cation MA_1–*x*_EA_*x*_PbI_3_ perovskite system. The hybrid compounds containing EA cations are gaining significant attention for their improved stability and photovoltaic performance.^24−29,42−44^ Pure EAPbI_3_ crystallizes into the orthorhombic (*Pnma*) symmetry^45−47^ with the anionic component having a 1D architecture composed of  columns which are built of face-sharing octahedra; however, no information is available on the structural phase transitions of this compound. The band gap of this compound is 2.2 eV, making it suitable for photovoltaic applications.^45^ In contrast, the phase transitions of pure MAPbI_3_ are rather well known: this compound exhibits two transitions at 327 and 162 K corresponding to the cubic  → tetragonal (*I*4/*mcm*) → orthorhombic (*Pnma*) symmetry lowering, followed by the MA cation ordering into a nonferroelectric phase.^17,48−51^

This work reveals that mixing MAPbI_3_ with EA stabilizes the cubic phase, as the temperatures of both structural phase transitions are significantly reduced. For a higher EA concentration, we obtain a complete suppression of the low-temperature phase transition and formation of a new tetragonal phase of a different symmetry. Our broad-band dielectric spectroscopy experiments of single-crystal samples indicate signatures of a dipolar glass phase and a substantial perturbation of the MA cation dynamics. Our experimental results are supported by density functional theory (DFT) calculations of the potential energy surface for molecular rotations. We also study the photoluminescence properties of these mixed compounds, revealing a small change of the band gap upon mixing.

## Experimental Details

### Sample Synthesis

Single crystals of MA_1–*x*_EA_*x*_PbI_3_ were grown in a similar way as recently proposed by Fateev et al. for crystallization of MAPbI_3_ and a number of 2D iodides.^52^ During a typical synthesis, 4 mmol of PbI_2_ and stoichiometric amounts of methylamine (2 M solution in methanol, Sigma-Aldrich) and ethylamine (2 M solution in methanol, Sigma-Aldrich) were added to a mixture of propylene carbonate (PC, 99.7%, Sigma-Aldrich) and HI (57 wt % in H_2_O, stabilized with H_3_PO_2_, Sigma-Aldrich) under stirring until complete dissolution of the solids (∼6 mL). The PC/HI volume ratio was 2.8:1, and the total amount of methylamine and ethylamine was 4 mmol. The clear solution was transferred into a glass vial with the lid slightly loosened. Then, the vial was kept at 50 °C for 2–3 days, and the grown black crystals with dimensions up to 5 mm were separated from the liquid and dried at room temperature. The crystals of EAPbI_3_ were yellow and up to 3 mm in size. The fraction *x* of the EA cations in MA_1–*x*_EA_*x*_PbI_3_ compounds was determined using ^1^H NMR spectroscopy.

### NMR Spectroscopy

^1^H NMR experiments were carried out at 14.1 T on a Bruker AVANCE Neo NMR spectrometer operating at 600.3 MHz using a 5 mm Bruker ^1^H-^13^C-BB TBI probe. The temperature was stabilized at 298 K. A pulse sequence employing 10 μs π/6 excitation pulse followed by 5 s repetition delay was employed, and 64 scans were accumulated. Samples were dissolved in DMSO-*d*_6_ (99.96% D atom, Sigma-Aldrich) which was used for lock. For signal assignment, the ^1^H–^1^H COSY pulse sequence was employed by collecting 4 scans for 256 increments. The obtained NMR spectra and their analysis are presented in the Supporting Information.

### Raman Spectroscopy

Room-temperature Raman spectra of polycrystalline samples were measured using a Bruker FT100/S spectrometer equipped with a YAG/Nd^3+^ laser excitation (1064 nm). The spectral resolution was set to 2 cm^–1^.

Temperature-dependent Raman spectra of randomly oriented single crystals of *x* = 0.38 and EAPbI_3_ samples were measured in the 1750–150 cm^–1^ range using a Renishaw inVia Raman spectrometer with a confocal DM2500 Leica optical microscope, a thermoelectrically cooled CCD detector, and a diode laser operating at 830 nm. Low-wavenumber Raman spectra of EAPbI_3_ (200–20 cm^–1^) were obtained using the same setup but with an extra Eclipse filter. A THMS600 temperature control stage for microscopy (Linkam) was used for temperature control in all experiments.

### Photoluminescence Spectroscopy

Temperature-dependent photoluminescence spectra were obtained in a closed cycle cryostat allowing measurements from 20 to 300 K. The *x* = 0.09, *x* = 0.16, and *x* = 0.21 samples were excited by a 532 nm line from a diode-pumped, solid-state laser (power, 500 μW), while a 405 nm laser (power, 500 μW) was used to excite the *x* = 0.38 sample. Additionally, the photoluminescence spectra were excited by a 325 nm line from a He–Cd laser. No significant differences in the photoluminescence spectra were observed for the three excitation wavelengths. Photoluminescence signals were collected using a Peltier-cooled Avantes CCD spectrometer.

### Differential Scanning Calorimetry

Differential scanning calorimetry (DSC) experiments of powder samples were performed using a Mettler Toledo DSC-1 calorimeter with a resolution of 0.4 μW. Nitrogen was used as a purging gas, and the heating/cooling rate was 5 K/min. Mass of the samples (in mg): 75.85 (*x* = 0), 69.99 (*x* = 0.09), 47.43 (*x* = 0.16), 31.02 (*x* = 0.21), 32.00 (*x* = 0.31), 29.76 (*x* = 0.38), and 32.45 (*x* = 1).

### Heat Capacity

Heat capacity at constant pressure *C*_*p*_ of all samples was measured in the temperature range of 1.8–350 K using thermal relaxation technique in the heat capacity option of the Physical Property Measurements System (PPMS). The typical accuracy of the system is better than 1% for temperatures above 100 K, and it slightly diminishes at lower temperatures. For the measurements, single-crystal samples were used with a typical sample mass of about 6 mg.

### Ultrasonic Measurements

The ultrasonic velocity and attenuation data of single crystal samples were obtained from the phase shift and amplitude of the received signal using a RITEC RAM-5000 pulse-echo ultrasonic measurement system. LiNbO_3_ transducers were used for the excitation and detection of the longitudinal ultrasonic waves at 10 MHz. The typical sample thickness was 2–3 mm, and the heating/cooling rate was kept below 1 K/min. Silicone oil was used between the sample, quartz buffer rods, and transducers to maintain acoustic bonds in the whole studied temperature range.

### X-ray Diffraction

Single-crystal X-ray diffraction (XRD) data were collected on the four-circle Xcalibur diffractometer operating with Mo Kα radiation, a CCD Atlas camera, and an Oxford diffraction cooling system. CrysAlisPro was used for the data processing (CrysAlis PRO 1.171.38.43 (Rigaku OD, 2015)). For the *x* < 1 samples, the lattice parameters were calculated for the archetype *Pm*3̅*m* cubic phase (using the orthorhombic restraints: all α, β, and γ angles were set to 90°).

Powder XRD (PXRD) measurements were performed on an X’Pert PRO powder diffractometer operating with Cu Kα radiation.

### Dielectric Spectroscopy

Broadband dielectric spectroscopy experiments of single crystal samples were performed in three different frequency bands. (i) 10 mHz to 100 kHz band: measurement of the capacitance and loss tangent with a Solartron ModuLab XM MTS system together with an XM MFA low-current module (only *x* = 0.38 sample). (ii) 20 Hz to 1 MHz band: measurement of capacitance and loss tangent with a HP4284A LCR meter (all samples). In both cases, the flat capacitor model was implemented to calculate the complex dielectric permittivity. (iii) 1 MHz to 1 GHz band: the complex reflection coefficient was measured with an Agilent 8714ET vector network analyzer. The multimode capacitor model was used to calculate the complex dielectric permittivity.^53,54^ Temperature-dependent dielectric spectra were measured on cooling at a rate of 1 K/min. In all cases, silver paste was used for sample electrodes. Temperature was measured with a Keithley Integra 2700 multimeter, a T-type thermocouple, and a 100 Ω platinum resistor. For measurements at low frequency (10 mHz to 100 kHz band), the temperature was stabilized before performing the experiment. The uncertainty of the sample size and imperfect calibration of the measurement setups result in about 3% error of the determined dielectric permittivity.

### DFT Calculations

The MA_0.875_EA_0.125_PbI_3_ mixed cation perovskite was simulated using DFT in the Vienna Ab initio Simulation Package (VASP) code.^55^ A 2 × 2 × 2 supercell in the settings of the parent cubic perovskite MAPbI_3_ was generated from the orthorhombic structure of MAPbI_3_, and all coordinates and cell parameters were relaxed. One of the eight MA cations was substituted with EA and fully relaxed. Several initial EA orientations within the same framework of MA orientations were tried, and the lowest energy one was selected for further modeling. Rotation barriers were constructed from the rigid rotation of the molecular cations around their center of mass. DFT calculations were performed with a plane-wave cutoff of 550 eV, a 4 × 4 × 4 Γ-centered *k*-point mesh, PAW pseudopotentials,^56^ and the PBEsol exchange–correlation functional.^57^

## Results and Discussion

We studied MA_1–*x*_EA_*x*_PbI_3_ perovskite crystals with the EA fraction of *x* = 0, 0.09, 0.16, 0.21, 0.31, 0.38, and 1, where *x* was determined by ^1^H NMR spectroscopy (see Figures S1 and S2, Supporting Information). We were not able to obtain the intermediate mixing levels (*x* > 0.4), indicating the EA solubility limit in MAPbI_3_ of about 40%, which is in a close agreement with the result reported by Wang et al.^58^ A subsequent sample characterization using room-temperature Raman spectroscopy also revealed a monotonous increase of the EA band intensity with increasing EA content (Figure S3), while the PXRD patterns exhibited no reflections of the secondary or unmixed phases (Figure S7). These experiments demonstrate successful incorporation of the EA cations in these compounds.

To study the behavior of the structural phase transitions of MA_1–*x*_EA_*x*_PbI_3_, we used a suite of different experimental techniques including measurements of heat capacity *C*_*p*_, DSC, ultrasonic propagation, dielectric properties, Raman spectroscopy, and single-crystal XRD. Such a combination of tools proved to be highly successful in distinguishing phase-transition anomalies from the experimental artifacts, as demonstrated by our previous studies on other mixed hybrid perovskite systems.^32,39^

**Figure 1:**
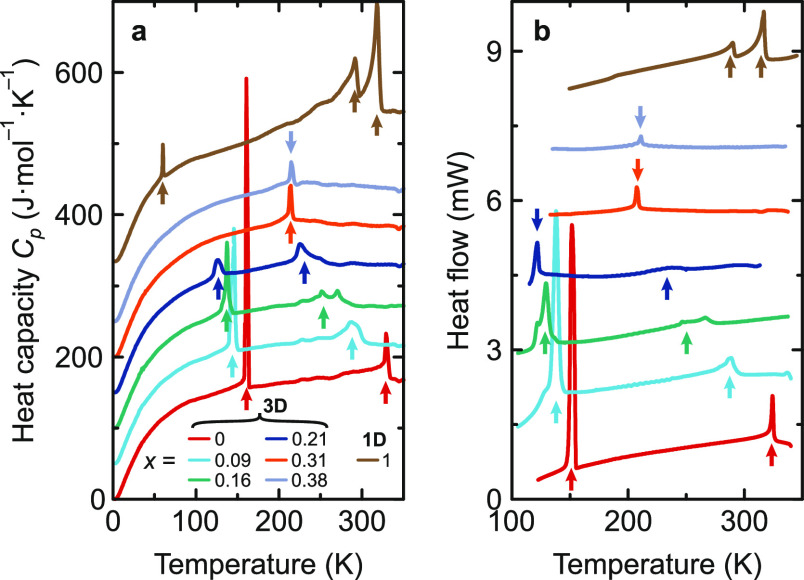
Temperature dependence of the (a) heat capacity and (b) DSC traces of MA_1–*x*_EA_*x*_PbI_3_ perovskites. Curves are offset by an arbitrary shift for clarity. Arrows indicate phase-transition anomalies.

The measured *C*_*p*_, DSC (Figure 1), and ultrasonic (Figure S8) data revealed two structural phase transitions of MAPbI_3_ (*x* = 0) at 329 and 161 K in agreement with previous reports.^17,48,50,51^ Upon increase of the EA fraction, we observed a gradual lowering of both transition temperatures, indicating stabilization of the desirable cubic phase. A similar behavior was also observed for the related mixed hybrid perovskites.^32,39,41^

**Figure 2:**
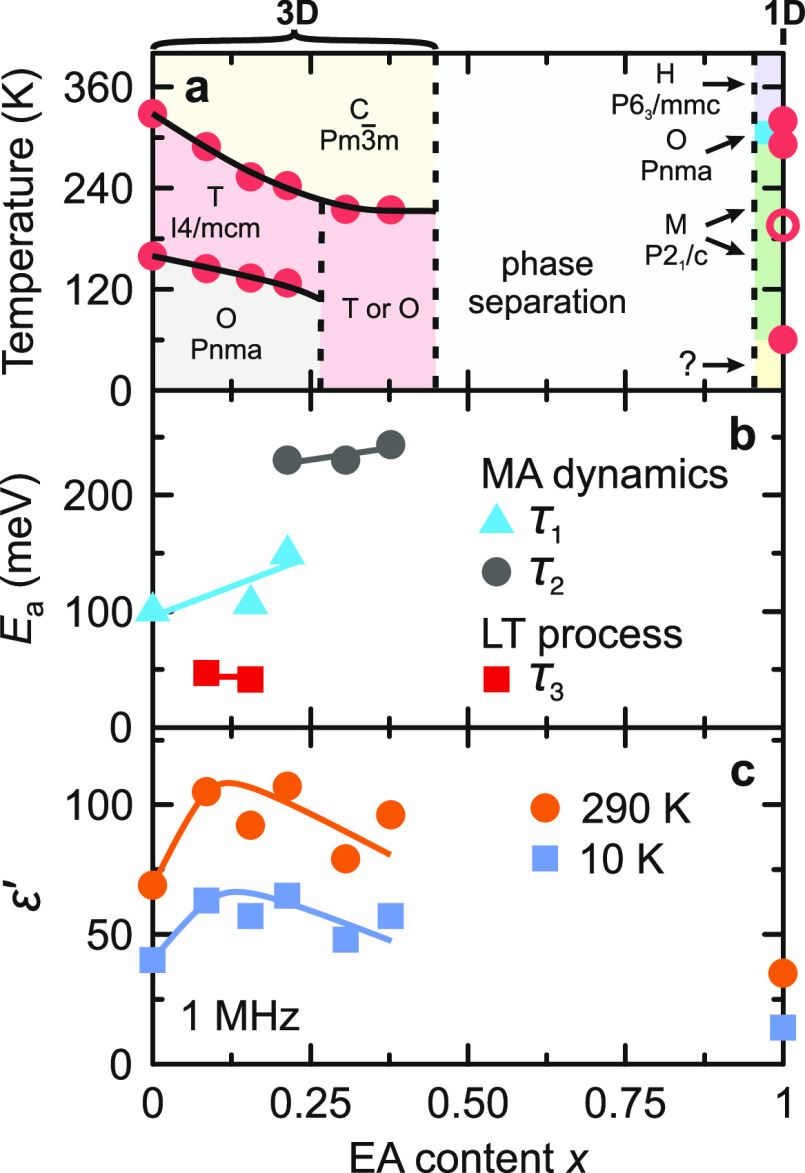
(a) Temperature–composition phase diagram of mixed MA_1–*x*_EA_*x*_PbI_3_ perovskites. Filled dots indicate structural phase transitions, while the empty dot marks the isostructural dielectric anomaly of EAPbI_3_. Dashed curves indicate tentative phase boundaries. Abbreviations: C—cubic, T—tetragonal, O—orthorhombic, H—hexagonal, and M—monoclinic. (b) EA concentration dependence of the activation energy of the MA cation dynamics and the low-temperature process. (c) ε′(1 MHz) vs. *x* obtained at 290 and 10 K. Solid curves are guide for the eye. Error bars are smaller than data points.

At higher concentrations of EA (*x* = 0.31 and 0.38), the low-temperature transition becomes completely suppressed (see Figures 1, S5, S8 and S9). Interestingly, the anomaly of the remaining transition is sharper compared to the lower EA concentrations. Our single-crystal XRD experiments also show that the space group of the *x* = 0.31 and 0.38 samples is no longer body-centered but instead can be described by a primitive tetragonal or pseudotetragonal (with very weak orthorhombic deformation) symmetry (see the Supporting Information). However, due to the pseudo-merohedral twinning, we were not able to obtain a reliable model of the low-temperature structure. Similar to undoped MAPbI_3_, the solution of this problem requires the use of a synchrotron or neutron radiation source for diffraction.^49,59^ We performed temperature-dependent Raman experiments to infer the low-temperature ordering of the *x* = 0.38 compound. Our experiments revealed that the Raman bands at 80 K of this sample are significantly wider compared to the nonmixed EAPbI_3_ (see Figures S4 and S6) or low-temperature structures of MAPbI_3_ and MAPbBr_3_,^60,61^ indicating a substantial disorder related to the molecular cations even at low temperatures. In particular, the tetragonal to orthorhombic phase transition in MAPbI_3_ and MAPbBr_3_ leads to drastic narrowing of the Raman bands near 1590 and 920 cm^–1^,^60,61^ which is not observed for the *x* = 0.38 sample, indicating the lack of MA ordering. We summarized the obtained results in the temperature–concentration phase diagram presented in Figure 2a.

We also performed the same type of experiments to study previously unknown structural phase transitions of EAPbI_3_, which crystallizes into a 1D perovskite structure.^47^ Our measurements revealed three phase-transition anomalies at 320, 292, and 60 K (Figures 1, S5, S8, S11). Interestingly, a transition at such a low temperature of 60 K is unusual for hybrid perovskites and related compounds and was never observed before.^20^ We used XRD experiments to study the symmetry lowering during the high-temperature transitions (see the Supporting Information) and obtained the hexagonal (*P*6_3_/*mmc*) → orthorhombic (*Pnma*) → monoclinic (*P*2_1_/*c*) decrease of symmetry on cooling (summarized in Figure 2a). Note that we did not study the low-temperature phase of EAPbI_3_, as the phase transition at 60 K is outside the temperature range accessible by our XRD equipment.

**Figure 3:**
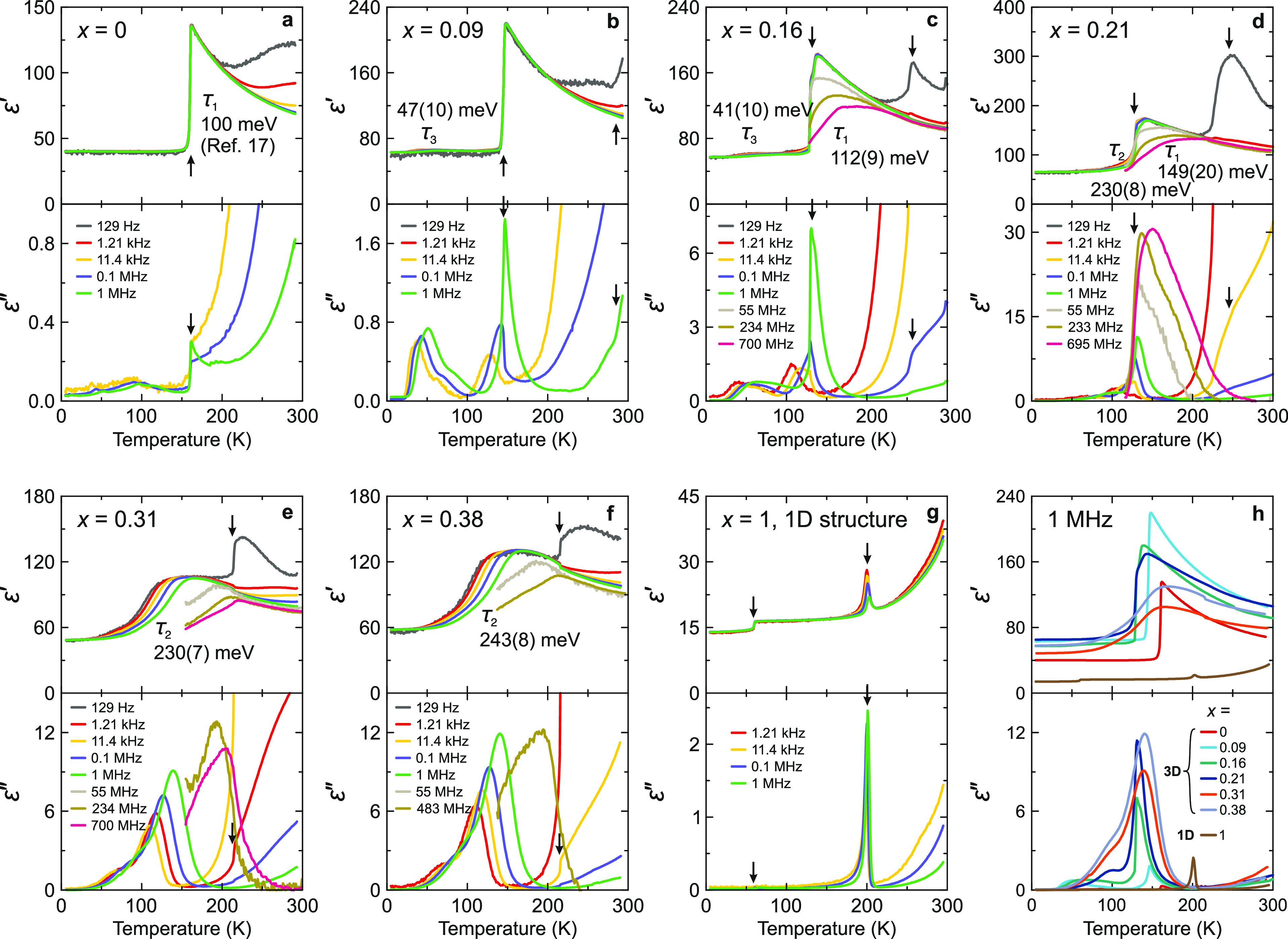
(a–g) Temperature dependence of the complex dielectric permittivity of MA_1–*x*_EA_*x*_PbI_3_ single crystals presented at selected frequencies. Arrows indicate phase-transition anomalies. Different relaxation processes (τ_1_, τ_2_, and τ_3_) are indicated together with the determined activation energies. (h) Comparison of the complex dielectric permittivity obtained at 1 MHz for all studied compositions.

We further performed broad-band dielectric spectroscopy experiments on the single-crystal samples to investigate the molecular cation dynamics in different regions of the phase diagram. The temperature dependence of the real ε′ and imaginary ε^″^ (dielectric loss) parts of complex dielectric permittivity ε* = ε′ – iε^″^ for all studied compositions is presented in Figure 3. For the *x* = 0 sample, ε* shows a sudden decrease at the tetragonal–orthorhombic phase transition point (Figure 3a), which is caused by the cooperative ordering of the MA electric dipoles.^17,32,39,41,62−64^ Upon mixing with the EA cations, the temperature of this anomaly decreases with increasing *x* (Figure 3b–d), in agreement with other experiments. For the highest mixing levels, the phase transition disappears transforming to a broad dipolar relaxation (Figure 3e,f). This indicates a substantial disorder at low temperatures in agreement with Raman spectroscopy results. Interestingly, for the *x* = 0 sample, the cubic–tetragonal phase transition exhibits no dielectric anomaly (Figure S11), but for the mixed compounds this transition becomes clearly visible in the low-frequency dielectric response (Figure 3b–f).

We also studied the dielectric properties of the nonmixed EAPbI_3_ compound (Figures 3g and S11). In addition to the aforementioned three phase transitions, we also observed a strong additional anomaly at about 190 K. Our XRD experiments revealed no associated symmetry change, which may indicate an isostructural character of this transition associated with the electric dipoles. A small anomaly at this temperature also appears in the Raman data (Figure S5). Note that such symmetry-preserving transitions are rather frequent in hybrid perovskites and related materials.^19,39,65^

Our dielectric experiments also allowed us to capture and distinguish different dipolar relaxation processes in the studied compounds. Based on previous studies, the rotational dynamics of the MA cations in the tetragonal phase of MAPbI_3_ occur in the GHz frequency range^66^ with the activation (barrier) energy *E*_a_ of 100 meV.^17^ We refer to this process as τ_1_. We performed sufficient broad-band experiments for the *x* = 0.16 and 0.21 samples, revealing the presence of this process (Figure 3c,d), while the analysis of the frequency domain data (see the Supporting Information) allowed the determination of *E*_a_ values: 112(9) meV (*x* = 0.16) and 149(20) meV (*x* = 0.21). This indicates that the MA rotation barrier increases with increasing concentration of the EA cations as is also summarized in Figure 2b. A similar trend and activation energies were also observed for other mixed perovskite systems,^32,39^ indicating a universal hindering of the MA motion upon mixing.

Due to the disappearance of the low-temperature transition in the highly mixed compounds (*x* = 0.31 and 0.38), the main dipolar relaxation extends to much lower temperatures (Figure 3e,f), and we refer to this process as τ_2_. Despite looking very similar to τ_1_, this relaxation has a much higher activation energy of *E*_a_ > 200 meV (Figures 2b and S13). Together with the complete suppression of the phase transition, this observation indicates a qualitatively different behavior of the molecular cations in this highly mixed region of the phase diagram. Note that this process is also very weakly visible just below the tetragonal–orthorhombic phase transition for the *x* = 0.21 sample (Figure 3d), suggesting that this concentration is somewhat intermediate between the two regimes.

Such a broad dipolar relaxation of the highly mixed compounds may indicate that the MA cations form a dipolar glass phase due to the dipole frustration introduced by mixing, which is frequently observed in mixed inorganic compounds.^67−70^ However, here, we did not observe a clear Vogel–Fulcher behavior of the mean relaxation time despite our broad-band (Hz–GHz) approach, as the Arrhenius law is sufficient to describe the obtained data (Figure S13). Observation of the Vogel–Fulcher law would indicate freezing of the electric dipoles and would provide an unequivocal evidence of such phase formation.^68,69^ The absence of this behavior suggests that the freezing might occur at very low temperatures. Note that similar hints to the glassy phase formation were also observed in the dielectric responses of the related MA_1–*x*_DMA_*x*_PbBr_3_ (strong signatures)^32^ and MA_1–*x*_FA_*x*_PbBr_3_ (weaker signatures)^39^ systems.

A third dipolar relaxation (τ_3_) is observed to occur in the weakly mixed compounds (*x* ≤ 0.16) at low temperatures (<100 K) (Figure 3b,c). The strength of this process and the activation energy (around 45 meV, Figure 2b) are much smaller compared to the τ_1_ and τ_2_ processes. There are also some indications of this process occurring in nonmixed MAPbI_3_, but we were not able to reliably extract the *E*_a_ value for this sample. Fabini et al.^62^ also detected a similar low-temperature relaxation for the MAPbI_3_ compound and assigned it to the dynamics of a glassy phase. On the other hand, Xu et al.^71^ used magnetic resonance to observe that the MA cations in MAPbI_3_ undergo twisting in the orthorhombic phase with the activation energy of 60 meV, which is rather close to the energy of the τ_3_ process determined from our experiments. As formation of a glassy phase in a nonmixed MAPbI_3_ compound is less likely, the second explanation seems more plausible.

EA cations also affect the value of dielectric permittivity of MA_1–*x*_EA_*x*_PbI_3_ (Figures 2c and 3). For small *x*, ε′ seems to slightly increase with increasing *x*, while for higher mixing levels it decreases, suggesting hindered dynamics of the MA cations. Note that the dielectric permittivity of the nonmixed EAPbI_3_ is much smaller compared to the mixed structures (Figure 3g,h). The value of ε′ obtained at 10 K, where cation relaxations are absent, is about 50 for all studied compounds except for EAPbI_3_ (ε′ ∼ 15). Similar values of the low-temperature dielectric permittivity were also obtained for other mixed lead halide perovskites.^32,39^ As discussed by Fabini,^72^ such a universal and rather high value of dielectric permittivity is related to the lattice polarizability induced by the 6s^2^ lone-pair electrons and associated off-centering of the lead cations.

**Figure 4:**
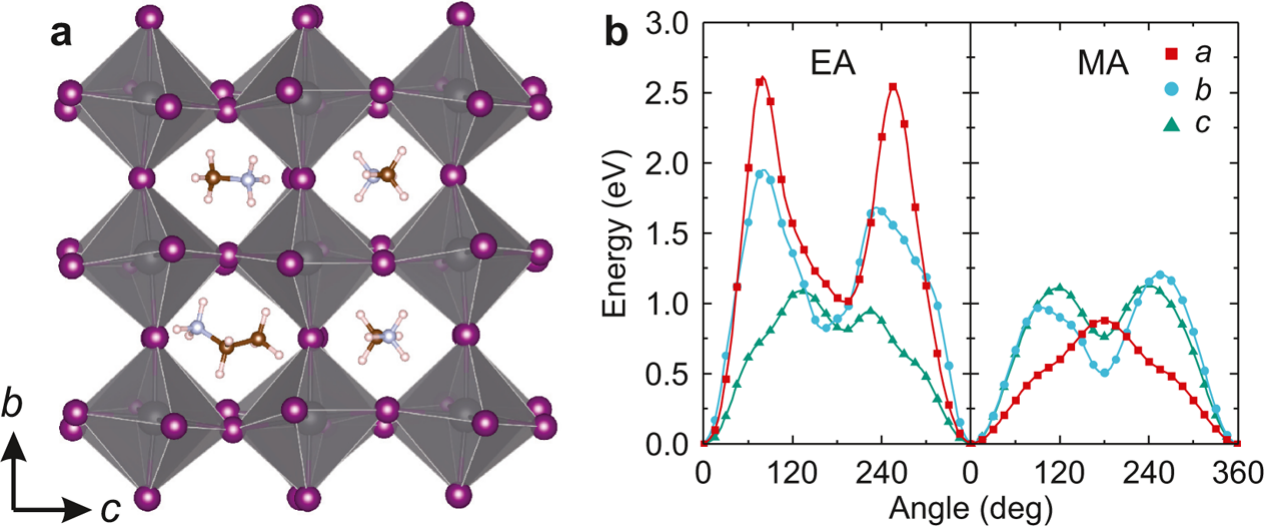
(a) Relaxed DFT structure of MA_0.875_EA_0.125_PbI_3_ used to calculate the rotation barriers of (b) the EA and the nearest-neighbor MA cation. For clarity, only a 2 × 2 × 1 slab containing the EA cation is presented. Curves are guides for eye.

To gain insights on the microscopic picture of mixing and to support our dielectric data, we performed DFT calculations on a mixed MA_0.875_EA_0.125_PbI_3_ supercell based on the orthorhombic structure of MAPbI_3_ (Figure 4a). Our calculations show that the long molecular axis (dipole moment) of the EA cation is approximately along the ⟨100⟩-family of crystal directions. A rotation potential was constructed by rotating the EA and the nearest-neighbor MA cation around three orthogonal lattice directions (Figure 4b). The obtained results indicate that the rotation barriers for the EA cations are about 2 times higher compared to the MA cations, except for the rotation axis, which coincides with the dipole moment of the EA cation and thus preserves its orientation. This strongly supports our claim that the dominant dielectric response originates from the MA cations.

We also used DFT calculations to assess the differences in the rotation barriers for crystallographically different MA cations in the MA_0.875_EA_0.125_PbI_3_ supercell. Our results indicate about 20% of variation in the rotational energies (see Figure S14), indicating that the dynamics of the MA cations are affected by the local lattice strains introduced by much bigger EA cations.^32^ It is likely that for a higher EA concentration, the MA rotation barriers would experience even stronger perturbations leading to a glassy behavior. Unfortunately, such DFT calculations would be very expensive, necessitating a different computational approach such as molecular dynamics.

**Figure 5:**
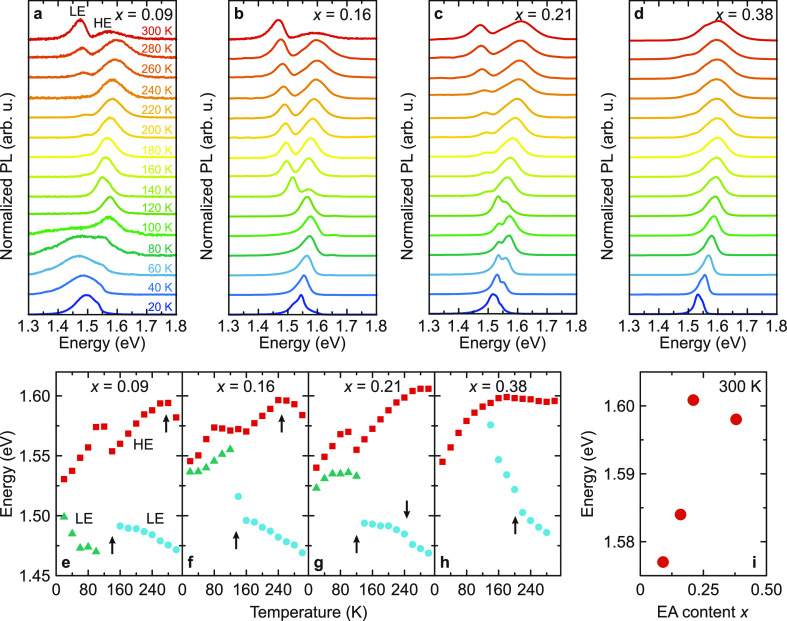
(a–d) Normalized photoluminescence spectra of MA_1–*x*_EA_*x*_PbI_3_ perovskites measured at different temperatures on cooling. (e–h) Temperature dependence of the LE and HE peak energies. Arrows indicate phase-transition points. (i) Energy of the free exciton emission at 300 K as a function of the EA content. Error bars are smaller than data points.

Finally, we also studied how cation mixing affects the optical properties of MA_1–*x*_EA_*x*_PbI_3_ compounds. Figure 5a–d shows the normalized temperature-dependent photoluminescence spectra of the *x* = 0.09, 0.16, 0.21, and 0.38 samples obtained under the same excitation conditions. For lower EA concentrations, two photoluminescence peaks of comparable amplitudes [low energy (LE) and high energy (HE)] can be clearly distinguished at room temperature, while the LE peak is significantly weaker for the highest mixing level. In general, the LE peak in hybrid perovskites may be attributed to bound excitons and defect states, while free excitons cause the HE emission.^46,73,74^

The temperature dependences of the HE and LE peak positions are indicated in Figure 5e–h, showing that the photoluminescence response is affected by the symmetry changes associated with structural phase transitions. For the *x* = 0.09, 0.16, and 0.21 samples, the LE peak seems to have the same nature in the cubic and tetragonal phases, while the temperature dependence is significantly different in the orthorhombic phase. The *x* = 0.38 compound exhibits negligible changes in the photoluminescence response at the phase-transition point, adding evidence for a different behavior of this highly mixed compound.

Comparison of the photoluminescence spectra and their decomposition into individual peaks (see Figure S15) revealed that the energy of the free exciton transition at room temperature slightly increases with increasing EA concentration and reaches saturation for the highest mixing level (Figure 5i). This can be attributed to widening of the band gap due to alloying with EAPbI_3_, which has a larger band gap,^45^ although changes in the exciton binding energy may also play a role. A slight increase of the bang gap of MA_1–*x*_EA_*x*_PbI_3_ with increasing *x* was also recently predicted by Liu et al.^42^

## Summary and Conclusions

We used a suite of different techniques to thoroughly study the structural phase transitions, phase diagram, cation dynamics, optical properties, and broad-band dielectric response of mixed-cation MA_1–*x*_EA_*x*_PbI_3_ hybrid perovskites. Understanding all these aspects is highly important for the successful applicability of novel EA-containing mixed hybrid perovskites in optoelectronics.

Our *C*_*p*_, DSC, and ultrasound experiments revealed a gradual lowering of the phase-transition temperatures upon mixing, indicating stabilization of the cubic phase. For higher-mixing levels (*x* ≳ 0.3), we observed a complete suppression of the low-temperature phase transition, while the XRD experiments revealed that in this highly mixed region the crystal symmetry changes from body-centered tetragonal to primitive tetragonal or weakly orthorhombic. Further studies involving more precise diffraction methods are necessary to probe this phase in more detail. As recently discussed by Huang et al. for the related MA_0.13_EA_0.87_PbBr_3_ perovskite,^44^ the disappearance of the low-temperature phase transition may also be related to the tolerance factor approaching unity upon mixing, which prevents large octahedral tilts associated with the orthorhombic *Pnma* phase. Upon further increase of *x*, we observed phase separation, indicating that the EA solubility limit in MAPbI_3_ is about 40%.

We used broad-band dielectric spectroscopy to study the molecular cation dynamics and dielectric properties of single-crystal compounds. Depending on the mixing level, three distinct dipolar relaxations were observed. For small *x*, the main relaxation occurs in the tetragonal phase, which we attribute to the rotational dynamics of the MA cations. Upon increase of the EA content, the activation energy of this process increases, indicating that the lattice deformation caused by the EA cations hinders the rotations of the MA cations. In the orthorhombic phase, a much weaker process is observed, which might be assigned to the twisting motion of the MA cations.

At the highest mixing levels (*x* = 0.21 and 0.38), we observed a broad dipolar relaxation extending to very low temperatures with a significantly higher activation energy compared to the weak mixed compounds. Such a relaxation indicates disordered phase and resembles a dipolar glass behavior. A very similar dipolar relaxation was also observed for other mixed hybrid perovskites,^32,39^ indicating a common behavior upon mixing, especially with the cations that cause a strong lattice distortion.

We also observed a substantial decrease of the dielectric permittivity value upon mixing, which might be related to much higher rotation barriers of the EA cations. The low-temperature permittivity value remains rather high (ε′ ∼ 50), indicating lattice polarizability by the lone-pair electrons of the lead cations,^72^ which also seems to be a universal behavior of the lead-based hybrid perovskites.

We supported our dielectric spectroscopy results with the DFT calculations of the moderately mixed *x* = 0.125 compound, which revealed that the rotation barriers of the EA cation are much higher compared to the smaller MA cations, indicating that the dominant dielectric response originates from the latter cations. Our calculations also indicate a considerable distribution of the MA cation rotation energies due to the local lattice strains introduced by the EA cations. This provides a microscopic mechanism behind the signatures of the glassy phase observed at the highest mixing levels.

Our temperature-dependent photoluminescence experiments revealed two emissions around 1.5 eV, which we attribute to the free and bound excitons. Both photoluminescence peaks are rather weakly affected by the structural phase transitions occurring in these compounds. We also observed a small increase of the emission energy of the free exciton with increasing EA content, which we attribute to the widening of the band gap.

## Supporting Information Available

Additional NMR, Raman, ultrasonic, XRD, dielectric, DFT, and photoluminescence data (PDF)
