# Supplementary material for: Mixology of MA1–xEAxPbI3 Hybrid Perovskites: Phase Transitions, Cation Dynamics, and Photoluminescence
Source: Chem Mater. 2022 Nov 2;34(22):10104–12. doi: 10.1021/acs.chemmater.2c02807 (PMC9686138; doi:10.1021/acs.chemmater.2c02807)
Supplement: Supplementary file 1 — cm2c02807_si_001.pdf [file cm2c02807_si_001.pdf]

Supporting Information

Mixology of $MA_{1-x}EA_xPbI_3$ Hybrid Perovskites: Phase Transitions, Cation Dynamics and Photoluminescence

Mantas Šimėnas,[*,†] Sergejus Balčiūnas,[†] Anna Gągor,[‡] Agnieszka Pieniążek,[¶] Kasper Tolborg,[§] Martynas Kinka,[†] Vytautas Klimavicius,[‖] Šarūnas Svirskas,[†] Vidmantas Kalendra,[†] Maciej Ptak,[‡] Daria Szewczyk,[‡] Artur P. Herman,[¶] Robert Kudrawiec,[¶] Adam Sieradzki,[⊥] Robertas Grigalaitis,[†] Aron Walsh,[§] Mirosław Mączka,[‡] and Jūras Banys[†]

†*Faculty of Physics, Vilnius University, Sauletekio 3, LT-10257 Vilnius, Lithuania*

‡*Institute of Low Temperature and Structure Research, Polish Academy of Sciences, Okólna 2, 50-422, PL-50-422 Wroclaw, Poland*

¶*Department of Semiconductor Materials Engineering, Wroclaw University of Science and Technology, Wybrzeze Wyspianskiego 27, PL-50-370 Wroclaw, Poland*

§*Thomas Young Centre and Department of Materials, Imperial College London, SW7 2AZ London, United Kingdom*

‖*Institute of Chemical Physics, Vilnius University, Sauletekio 3, LT-10257 Vilnius, Lithuania*

⊥*Department of Experimental Physics, Wroclaw University of Science and Technology, Wybrzeze Wyspianskiego 27, PL-50-370 Wroclaw, Poland*

E-mail: mantas.simenas@ff.vu.lt

Additional Experimental Data

^{1}H NMR spectroscopy data

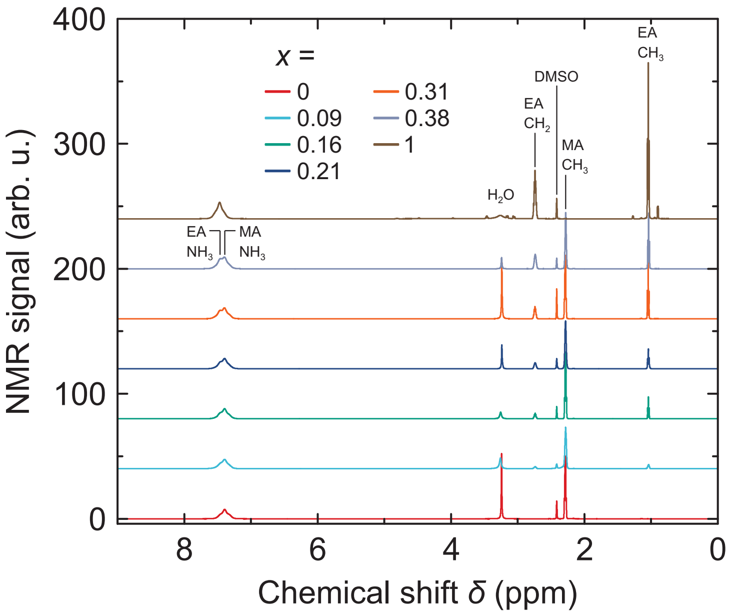

Figure S1: Stacked room temperature ^{1}H NMR spectra of $MA_{1\text{-}x}EA_xPbI_3$ used to determine the fraction of the EA cations in the synthesized compounds. The spectra for $x < 1$ are normalized to the MA cation methyl group signal. The signal assignment is based on the NMR COSY spectrum (see Figure S2).

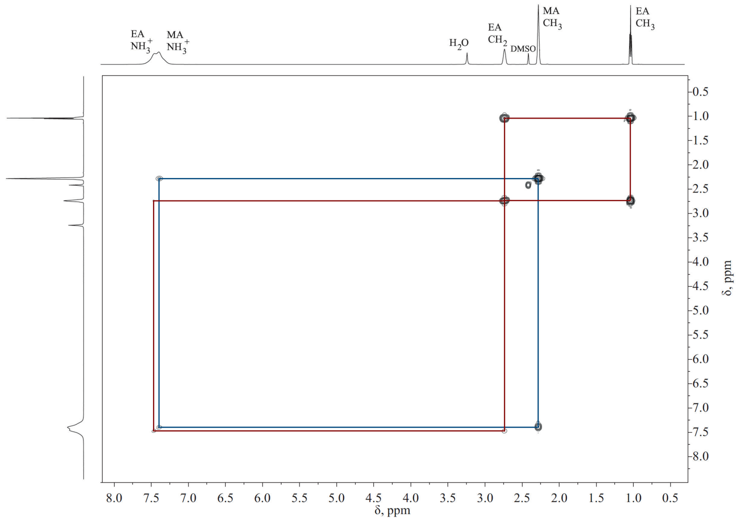

Figure S2: Room temperature ^{1}H NMR COSY spectrum of $MA_{0.62}EA_{0.38}PbI_3$. The cross peaks confirm our assignment of the ^{1}H NMR signals.

Raman spectroscopy data

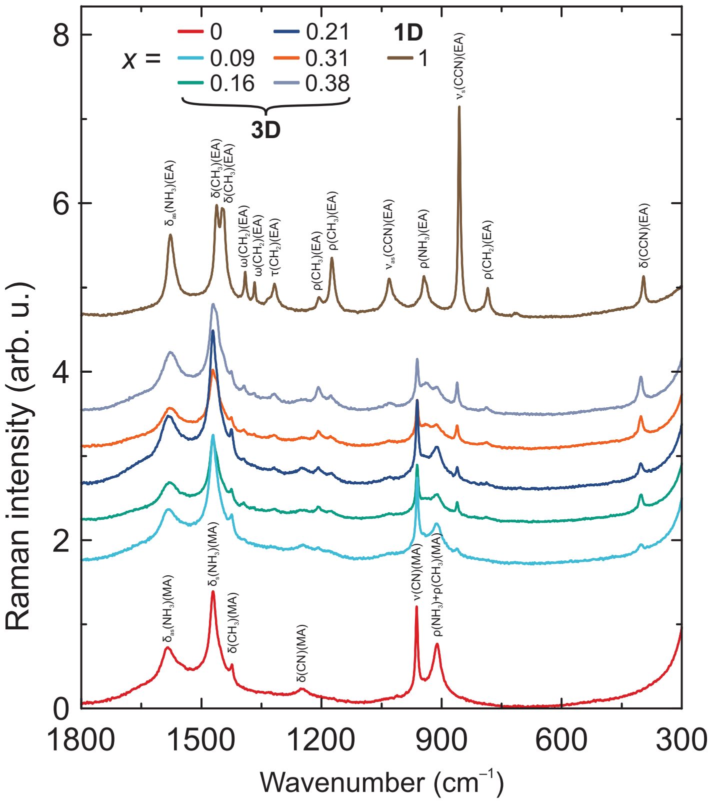

Figure S3: Stacked room temperature Raman spectra of $MA_{1-x}EA_xPbI_3$ with the corresponding assignment of the molecular vibrations based on the previous studies.[1,2]

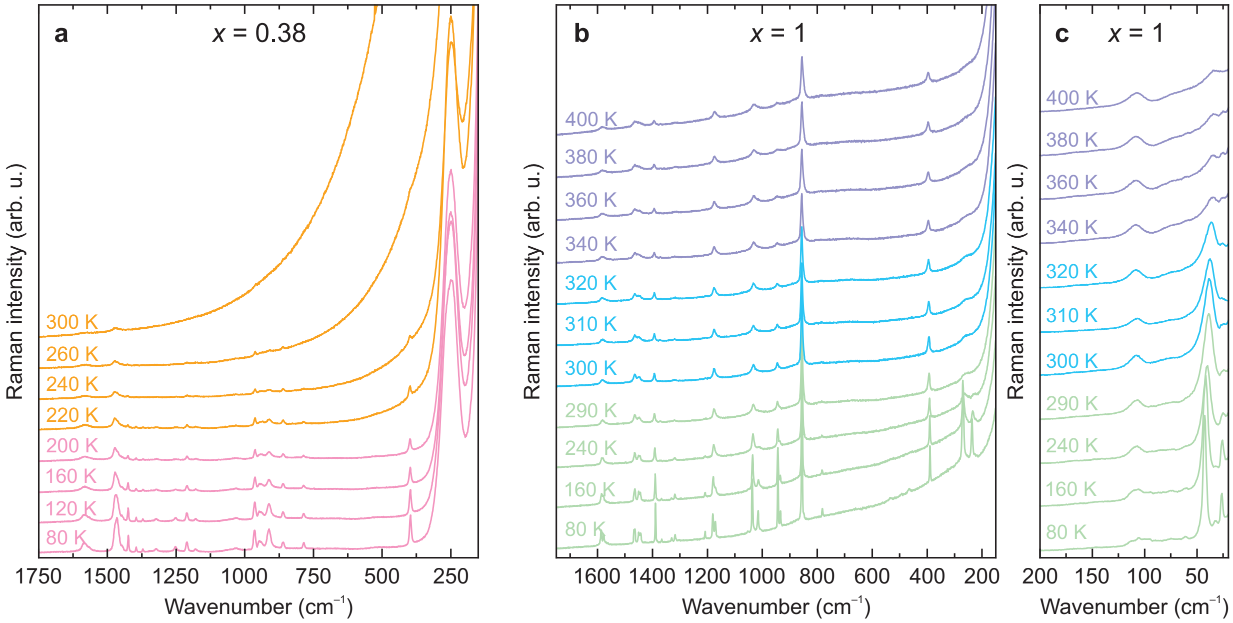

Figure S4: Temperature-dependent Raman spectra of the (a) $x = 0.38$ sample measured in the $1750-200$ cm^{-1} range compared to the $x = 1$ sample measured in the (b) $1750-150$ cm^{-1} and (c) $200-20$ cm^{-1} ranges.

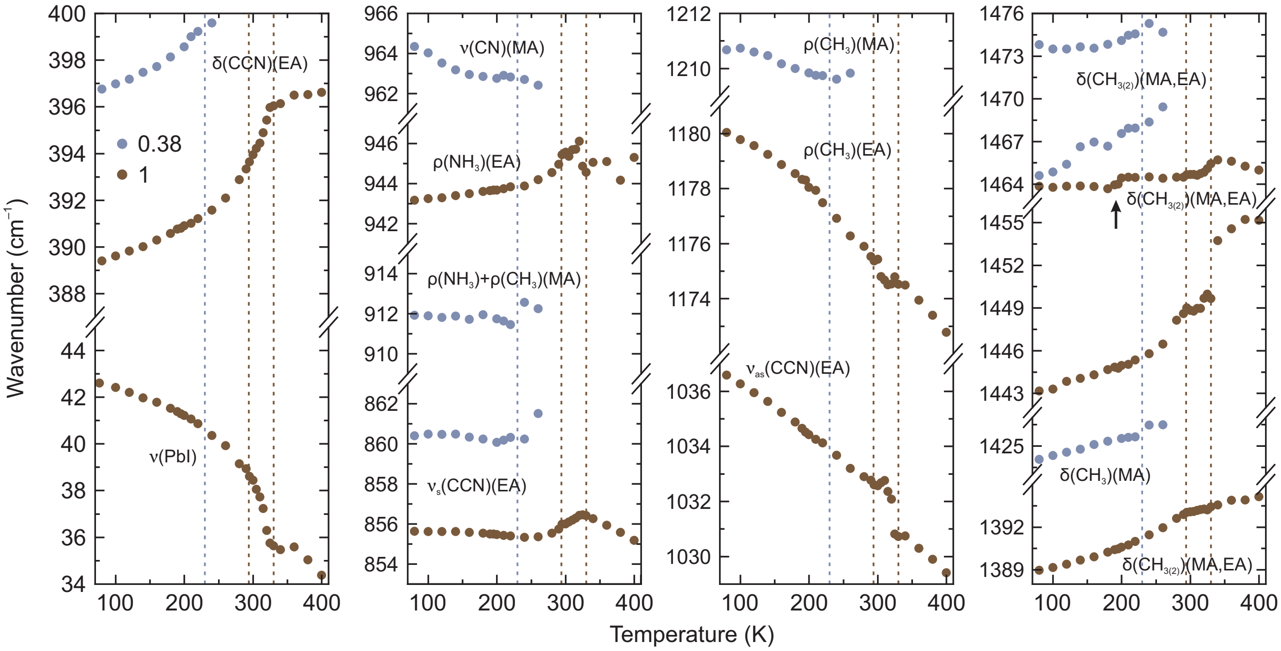

Figure S5: Thermal evolution of selected Raman bands for the $x = 0.38$ and $x = 1$ samples. The vertical lines correspond to temperatures of the phase transitions, while the arrow indicates the most pronounced anomaly due to the isostructural transition of $EAPbI_3$.

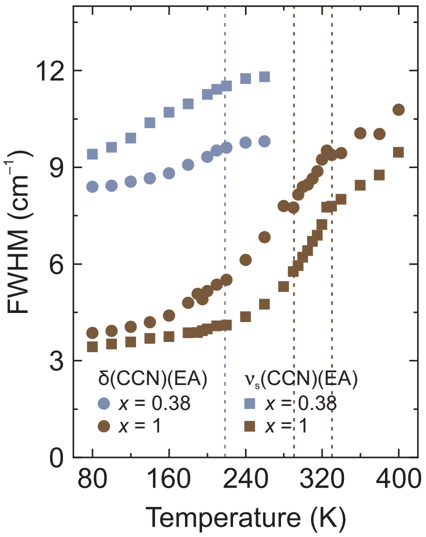

Figure S6: Temperature dependence of the selected Raman bandwidths of the $x = 0.38$ and $x = 1$ samples. Significantly wider Raman bands of the mixed compound compared to $EAPbI_3$ indicate presence of disorder at low temperature. The vertical lines correspond to temperatures of the phase transitions.

Powder XRD data

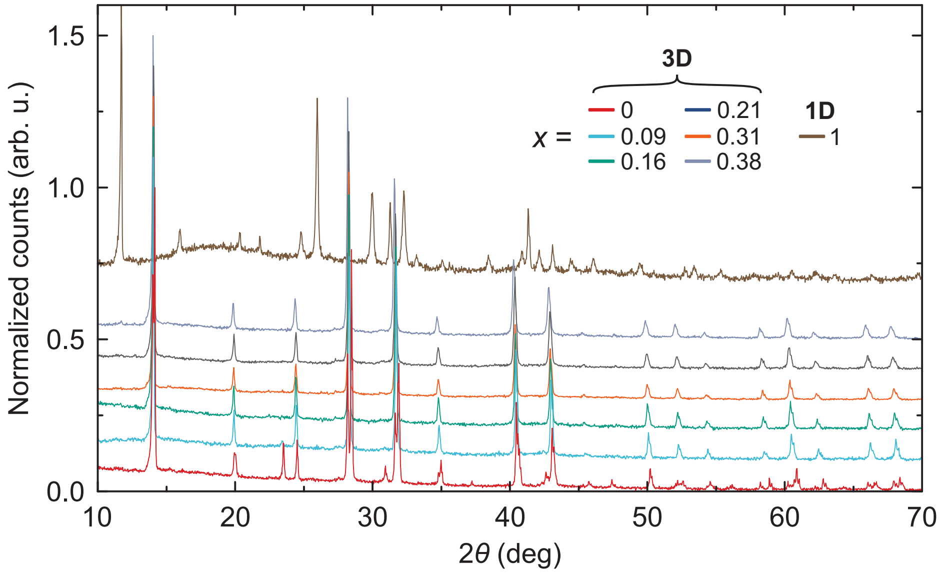

Figure S7: Stacked room temperature powder XRD patterns of $MA_{1-x}EA_xPbI_3$.

Ultrasonic data

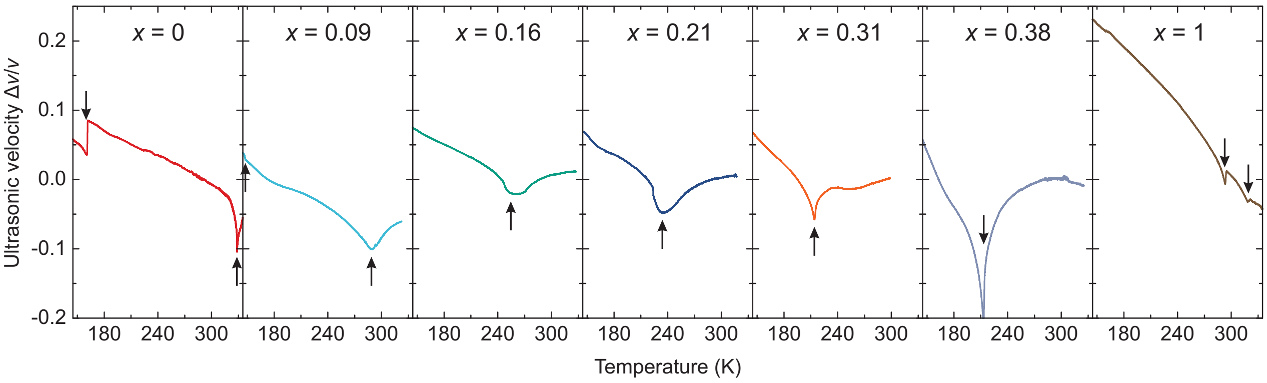

Figure S8: Temperature dependence of the ultrasonic velocity of $MA_{1-x}EA_xPbI_3$ single crystal samples. The change of the ultrasonic velocity is normalized to the room temperature value. The arrows indicate phase transition anomalies.

Single crystal XRD

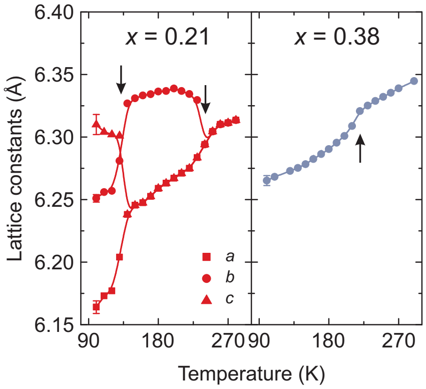

Figure S9: Temperature dependence of the lattice parameters of $MA_{1\text{-}x}EA_xPbI_3$ ($x = 0.21$ and 0.38) single crystal samples. The arrows indicate phase transition anomalies. A pseudo cubic model was used to refine the structures of $x = 0.38$ composition. The error bars are indicated on the lowest temperature points.

Reciprocal space reconstructions for $x = 0.21$ and $x = 0.38$ samples are presnted in Figure S10. The obtained diffraction patterns for the $x = 0.21$ sample are characteristic of tetragonal I-centered and primitive orthorhombic P lattice at 175 K and 110 K, respectively. They fit the phase diagram of $MAPbI_3$, where the symmetry reduction from $Pm\bar{3}m$ to $I4/mcm$, and to $Pnma$ is observed.[3] For the $x = 0.38$ sample, the diffraction picture is different. The patterns at 170 K do not fit the I-centered unit cell, whereas those at 120 K still resemble pseudo-merohedral twins, which in $MAPbI_3$ are present in the tetragonal phase. Therefore, a likely scenario is the formation of a new, tetragonal, or pseudo-tetragonal low-temperature phase for the highest concentration of EA in $MA_{1\text{-}x}EA_xPbI_3$ system.

Table S1 presents the crystal data, data collection and refinement results of the hexagonal and monoclinic structures of $EAPbI_3$. The detailed structural information is given in crystallographic information files (cif files) deposited in CCDC database with deposition numbers 2189712 and 2189713.

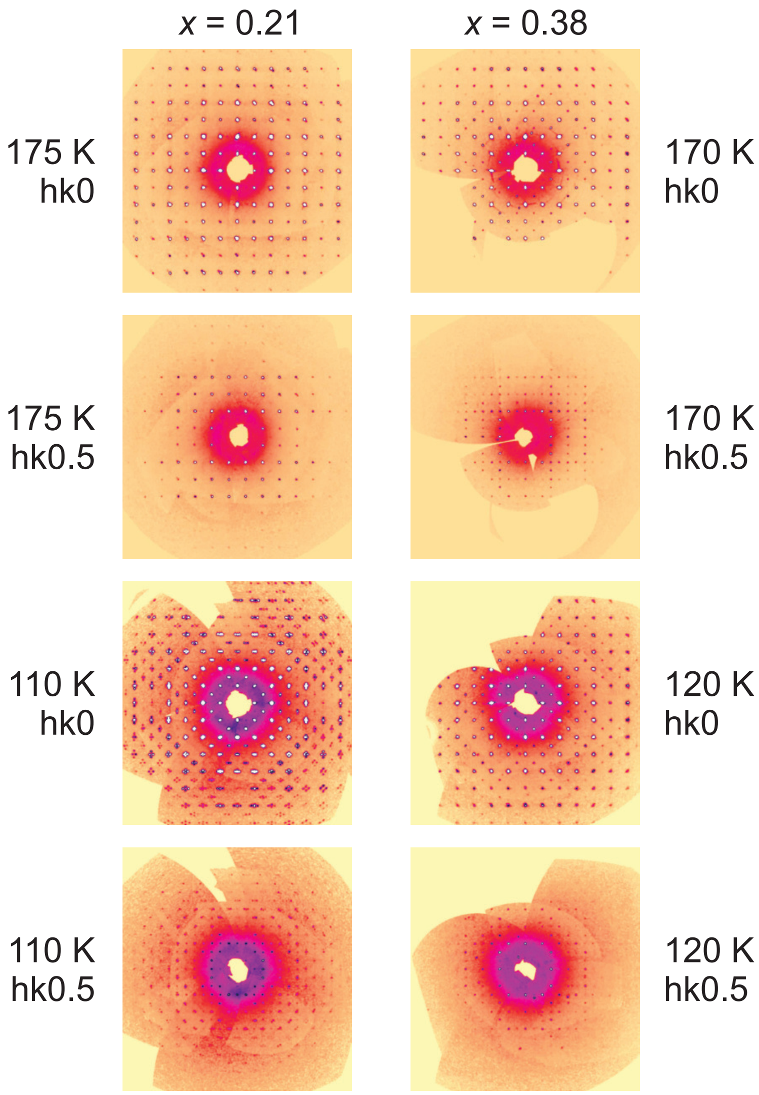

Figure S10: Reciprocal space reconstructions for $x = 0.21$ and $x = 0.38$ samples at different temperature. The Miller indexes are set according to the high-temperature cubic phase.

Table S1: Crystal data, data collection and refinement results of the hexagonal and monoclinic structures of $EAPbI_3$

	**Phase I**	**Phase III**
**Crystal data**		
Chemical formula	$I_3Pb \cdot C_2H_8N$	$I_3Pb \cdot C_2H_8N$
M_r	633.98	633.98
Crystal system, space group	Hexagonal, $P6_3/mmc$	Monoclinic, $P2_1/c$
Temperature (K)	340	120
a, b, c (Å)	8.8310(9), 8.8310(9), 8.0694(11)	8.5854(7), 14.7501(15), 8.1378(9)
α, β, γ (°)	90, 90, 120	90, 93.716(9), 90
V (Å^3)	544.99(13)	1028.37(18)
Z	2	4
μ (mm^{-1})	23.90	25.34
Crystal size (mm)	0.18 × 0.12 × 0.07	0.18 × 0.12 × 0.07
**Data collection**		
T_{min}, T_{max}	0.398, 1.000	0.692, 1.000
No. of measured, independent and observed $[I > 2\sigma(I)]$ reflections	4131, 311, 249	4044, 4044, 3480
R_{int}	0.030	-
$(\sin\theta/\lambda)_{max}$ (Å^{-1})	0.692	0.689
**Refinement**		
$R[F^2 > 2\sigma(F^2)], wR(F^2), S$	0.036, 0.102, 1.16	0.037, 0.099, 1.04
No. of reflections	311	4044
No. of parameters	11	67
No. of restraints	2	0
H-atom treatment	–	H-atom parameters constrained
$\Delta >_{max}, \Delta >_{min}$ (e Å^{-3})	0.76, -1.12	3.44, -3.47

Computer programs: CrysAlis PRO 1.171.38.41 (Rigaku OD), SHELXT 2014/5,[4] SHELXL 2018/3,[5] Olex2 1.5.[6]

Additional dielectric spectroscopy data

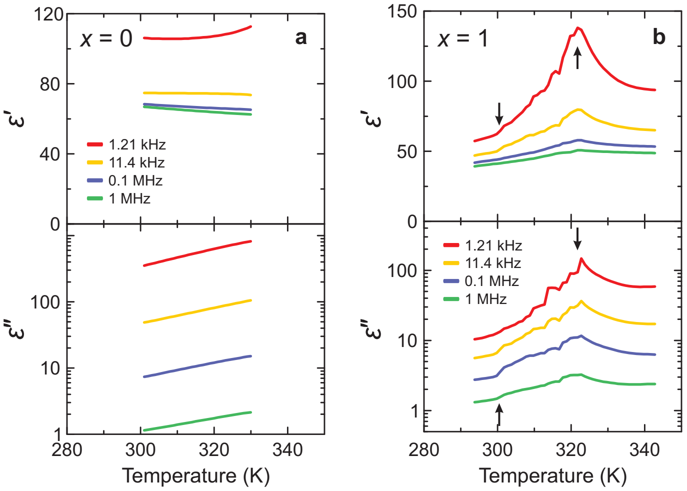

Figure S11: Temperature dependence of the complex dielectric permittivity of (a) $MAPbI_3$ and (b) $EAPbI_3$ single crystals in the high temperature region obtained on heating. The arrows mark the phase transition anomalies. The cubic-tetragonal phase transition of $MAPbI_3$ is not resolved in the dielectric data.

The frequency dependences of ε^* of $MA_{1\text{-x}}EA_xPbI_3$ compounds ($x = 0.21$, 0.31 and 0.38) are presented in Figure S12 for selected temperatures. The observed relaxations correspond to the main MA cation dynamics (τ_1 ($x = 0.21$) and τ_2 ($x = 0.31$ and 0.38) process). The relaxations were approximated using a Cole-Cole equation:[7]

$$\varepsilon^*(\omega) = \varepsilon(\infty) + \frac{\Delta\varepsilon}{1 + (i\omega\tau)^{1-\alpha}}. \tag{S1}$$

Here $\varepsilon(\infty)$ is the dielectric permittivity in the high-frequency limit, $\Delta\varepsilon$ denotes the dielectric strength of the process, τ is the mean relaxation time, and $\omega = 2\pi\nu$ is the angular measurement frequency. The relaxation width is described the parameter $0 \leq \alpha < 1$. For $\alpha = 0$, the Cole-Cole process reduces to the Debye relaxation, which describes non-interacting electric dipoles.

The best fits of Eq. S1 to the frequency domain data are also presented in Figure S12. For $x = 0.21$, the determined value of α at 110 K is around 0.53(1), while it increases to 0.80(1) for the $x = 0.38$ sample indicating much broader relaxation for the highly mixed

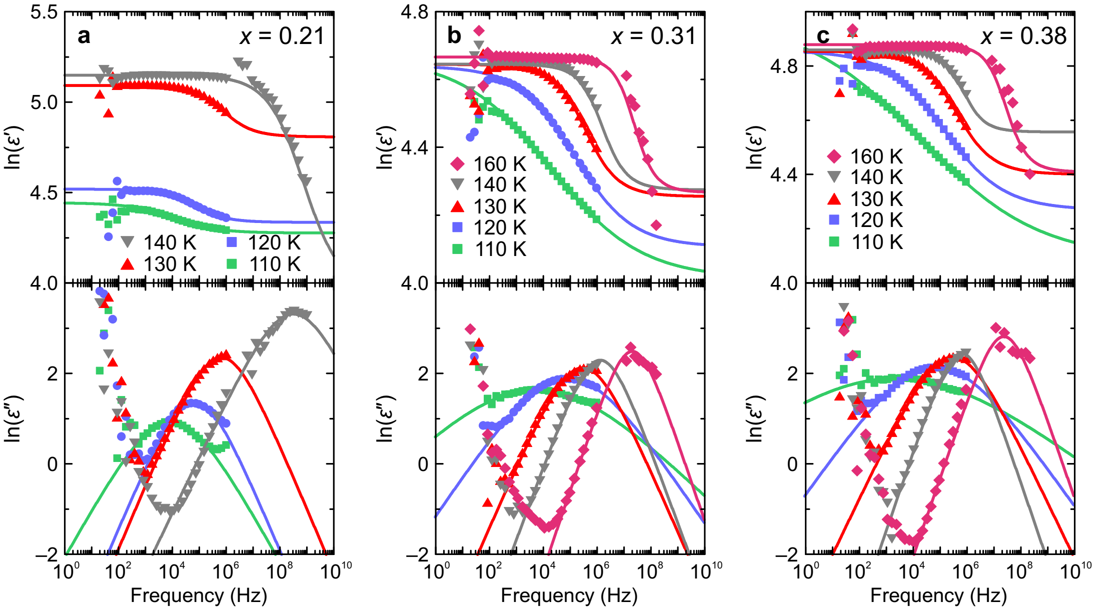

Figure S12: Frequency dependence of the complex dielectric permittivity of $MA_{1\text{-}x}EA_xPbI_3$ ($x = 0.21$, 0.31 and 0.38) single crystal compounds presented at selected temperatures. The solid curves are the best fits to a single Cole-Cole relaxation process.

compound.

The obtained temperature dependences of the mean relaxation times τ of all observed processes are presented in Figure S13. For all studied compounds, the relaxation times follow the Arrhenius law: $\tau = \tau_0 \exp(E_a/kT)$, where E_a and τ_0 denote the activation energy and attempt time, respectively, and k is the Boltzmann constant. The determined activation energies are also presented in Figure S13.

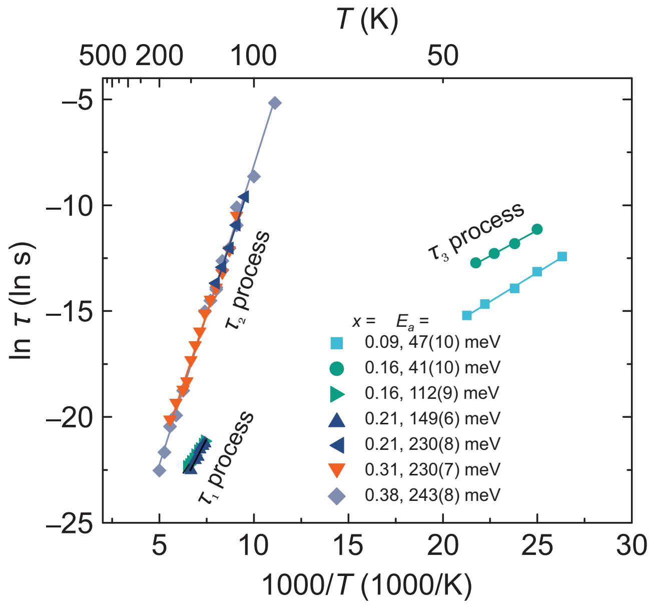

Figure S13: Inverse temperature dependence of the mean relaxation time of different processes observed in $MA_{1-x}EA_xPbI_3$ compounds measured by the broadband dielectric spectroscopy. The solid curves indicate fits to the Arrhenius equation.

Additional DFT data

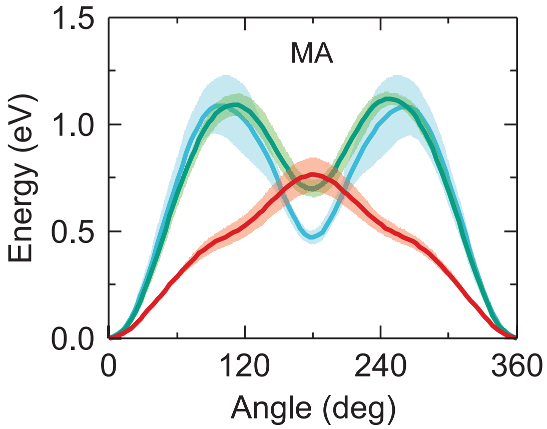

Figure S14: Averaged rotation barriers of the MA cations in $MA_{0.875}EA_{0.125}PbI_3$ supercell calculated by the DFT. The shaded regions mark the standard deviation of the rotation barriers.

Additional photoluminescence data

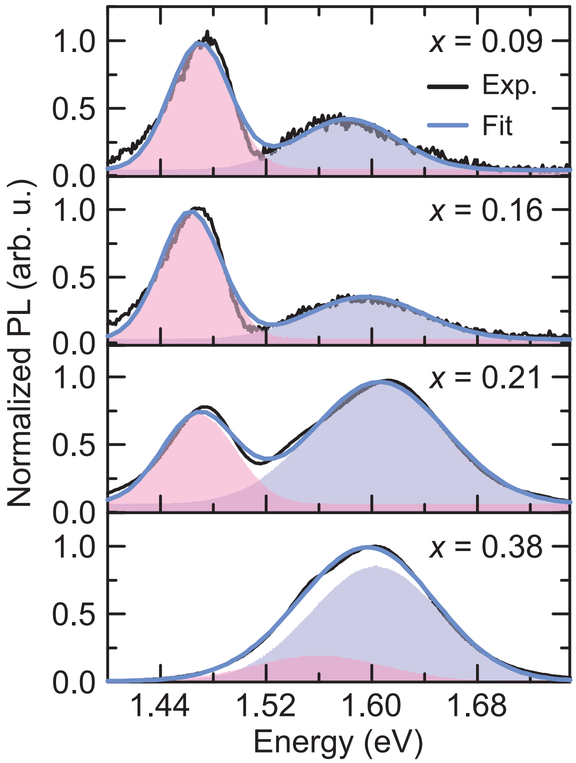

Figure S15: Normalized PL spectra of $MA_{1-x}EA_xPbI_3$ samples measured at 300 K and fitted by two Gaussian peaks: the high energy peak is attributed to free exciton emission, while the low energy peak may be attributed to bound excitons.

References

(1) Zeroka, D.; Jensen, J. O.; Samuels, A. C. Infrared Spectra of Some Isotopomers of Ethylamine and the Ethylammonium Ion: a Theoretical Study. *J. Mol. Struct.: THEOCHEM* **1999**, *465*, 119–139.

(2) Ptak, M.; Mączka, M.; Gagor, A.; Sieradzki, A.; Stroppa, A.; Di Sante, D.; Perez-Mato, J. M.; Macalik, L. Experimental and Theoretical Studies of Structural Phase Transition in a Novel Polar Perovskite-Like $[C_2H_5NH_3][Na_{0.5}Fe_{0.5}(HCOO)_3]$ Formate. *Dalton Trans.* **2016**, *45*, 2574–2583.

(3) Whitfield, P. S.; Herron, N.; Guise, W. E.; Page, K.; Cheng, Y. Q.; Milas, I.; Crawford, M. K. Structures, Phase Transitions and Tricritical Behavior of the Hybrid Perovskite Methyl Ammonium Lead Iodide. *Sci. Rep.* **2016**, *6*, 35685.

(4) Sheldrick, G. M. *SHELXT* – Integrated Space-Group and Crystal-Structure Determination. *Acta Crystallogr. A* **2015**, *71*, 3–8.

(5) Sheldrick, G. M. Crystal Structure Refinement with *SHELXL*. *Acta Crystallogr. C* **2015**, *71*, 3–8.

(6) Dolomanov, O. V.; Bourhis, L. J.; Gildea, R. J.; Howard, J. A. K.; Puschmann, H. *OLEX2*: a Complete Structure Solution, Refinement and Analysis Program. *J. Appl. Crystallogr.* **2009**, *42*, 339–341.

(7) Schonhals, A.; Kremer, F. *Broadband Dielectric Spectroscopy*, 1st ed.; Springer-Verlag Berlin Heidelberg, 2003.
